# A new switching parameter varying optoelectronic delayed feedback model with computer simulation

**DOI:** 10.1038/srep22295

**Published:** 2016-02-29

**Authors:** Lingfeng Liu, Suoxia Miao, Mengfan Cheng, Xiaojing Gao

**Affiliations:** 1 School of Software, Nanchang University, Nanchang, 330031, PRC; 2 Faculty of Science, Nanchang Institute of Technology, Nanchang, 330029, PRC; 3 School of Optical and Electronic Information, Huazhong University of Science & Technology, Wuhan, 430074, PRC; 4 School of Automation, Huazhong University of Science & Technology, Wuhan, 430074, PRC

## Abstract

In this paper, a new switching parameter varying optoelectronic delayed feedback model is proposed and analyzed by computer simulation. This model is switching between two parameter varying optoelectronic delayed feedback models based on chaotic pseudorandom sequences. Complexity performance results show that this model has a high complexity compared to the original model. Furthermore, this model can conceal the time delay effectively against the auto-correlation function, delayed mutual information and permutation information analysis methods, and can extent the key space, which greatly improve its security.

Optical chaos systems have superior advantages of generating wide-frequency spectrum, low attenuation and high transmission rate, and thus raising wide attention of researchers and engineers^1^,^2^,^3^. At present, one of the two major methods to generate optical chaotic signal is based on the inner nonlinear effects of semiconductor lasers^4^,^5^,^6^ including all-optical feedback, optical injection and optoelectronic feedback. Theoretically, these systems can be characterized by the rate equations. Another method is based on the nonlinear effects of external nonlinear devices, among which Mach-Zehnder modulator (MZ) may be the most common device used in this implementation^7^,^8^,^9^,^10^. The optoelectronic delayed feedback system based on MZ modulator has high modulation speed, good stability, large Lyapunov dimension, low cost in experimental and predominance in optical chaos communications. These great advantages make it receive extensively investigated. In theory, this second method can be modeled by Ikeda’s delay differential equations.

Although the optoelectronic delayed feedback system based on MZ modulator are proved to have high dynamical complexity, many studies also show that these systems are still vulnerable to attackers^11^,^12^, which are not suitable for some applications, especially for secure communications. Optical chaos systems have two major security deficiencies that the key space is not large enough to resist brute-force attacks^13^ and that the output signals carry certain characteristics of the original optical systems, causing time-delay disclosure. Currently, five alternative methods have been proposed to detect the time-delay, including auto-correlation function (ACF)^13^,^14^, delayed mutual information (DMI)^15^, permutation information analysis (PIA)^16^, extrema statistics^17^, and filling factor^18^. Among these methods, ACF, DMI and PIA are the most effective due to non-sensitiveness to noise.

The most effective method treat the first deficiency is to increase the number of tunable parameters in the system^19^,^20^. To remedy the second shortcoming, the most widely used method is to adjust the parameter of original optical system. Generally, three kinds of parameters can be selected, including time-delay^21^,^22^, gain coefficient^23^ and phase^24^. Among them, varying the phase needs to generate a pseudorandom number sequence with a generation rate no less than 3Gb/s, which is difficult to implement. Therefore, varying time-delay or gain coefficient may be the most useful and common methods to conceal the time-delay.

In this paper, we propose a new switching parameter varying optoelectronic delayed feedback model by computer simulation. Our model is to switch between two parameters varying optoelectronic delayed feedback models based on chaotic pseudorandom sequences. Based on digital chaotic map, we vary the gain coefficient of one sub-model and the time-delay of the other sub-model. The trajectory diagram, bifurcation diagram, approximate entropy (ApEn) and permutation entropy (PE) are used for illustrate the model performance. Results indicate that this model has a high complexity compared to the original systems in ref. 9. Thus we conclude that our new model can reduce the time delay more effectively compare to the ACF, DMI and PIA methods. Furthermore, tunable parameters can be used to extent the key space to resist brute-force attacks. This new model has varies applications in many fields such as computer secure communication, random sequence generator and cryptography.

The rest of this paper is organized as follows. In Section 2, the new switching model is introduced. The complexity performance of this model is introduced in Section 3. The time-delay concealment is analyzed in Section 4. Finally, Section 5 concludes the whole paper.

**New switching parameter varying optoelectronic delayed feedback model**. The original optoelectronic delayed feedback system in ref. 9 can be modeled by the following integro-differential delayed equation:


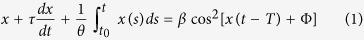


where *β = πgAGP/*2*V*_*π*_, Φ = *πV*_*B*_/2*V*_*dc*_, *θ* = 1/2*πf*_*L*_, and *τ* = 1/2*πf*_*H*_. *V*_*π*_ and *V*_*dc*_ are radio-frequency and dc half-wave voltages of MZ modulator; *V*_*B*_ is the biased voltage; *P* is the power of semiconductor laser source; *T* is the time-delay; *g* and *G* are the gain coefficients of photodiode and ratio-frequency amplifier, respectively. *A* is the overall attenuation of feedback loop; *f*_*L*_ and *f*_*H*_ are low and high cutoff frequencies, respectively.

Model (1) has been proved to be unsecure^21^,^23^. Here, we propose a new switching model with varying parameters, which can be written as:


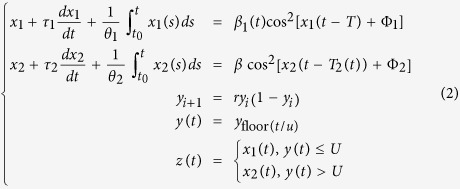


where *x*_1_ and *x*_2_ are the output signals of sub-model 1 and 2. *τ*_1, 2_, *θ*_1, 2,_, Φ_1, 2_, *T*, *T*_2_(*t*), *β*_1_(*t*) and *β* are the parameters of the two sub-models, respectively. *r* is the control coefficient of the logistic map with output *y*_*i*_. *U* is the switching threshold. *u* is the time interval, and operating floor (*t*/*u*) changes the completely discrete signal *y*_*i*_ into signal *y*(*t*). *y*(*t*), a step signal with time interval *u,* is not continuous. *z*(*t*) is the final output signal of this model.

As for sub-models 1 and 2 shown in Eq. (2), the parameters of sub-models 1 and 2 are both varying. For sub-model 1, the gain coefficient *β* is varying; and for sub-model 2, the time-delay *T* is varying. The varying strategy is as follows.


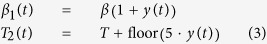


Also, *β*(*t*) and *T*(*t*) are modified every *u* time rather than continuously. *β*(*t*) and *T*(*t*) will remain the same when *ku* ≦* t* < (*k* + 1)*u*, (*k* = 0, 1, 2, …). In Eq. (3), 5 refers to 5 ns.

As explained ealier, the transfer frequency of output signal *z*(*t*) and the parameter varying signal *β*(*t*), *T*(*t*) are all determined by time interval *u*. The larger the time *u* is, the slower transfer frequency is take *u* = 5 ns as an example, set *r* = 3.9999, *y*_0_ = 0.1, *U* = 0.5, *β* = 4.5, *T* = 30 ns, the switching signal *z*(*t*) and parameter varying signal *β*(*t*), *T*(*t*) are shown in Fig. 1.

Evidently, the tunable parameters of this new model include *β*, *T*, *r*, *U* and three initial values. Compare to the original model (1), the parameter *r* and initial value *y*_0_ of digital chaotic map are newly introduced. Assume the precision is 10^−16^, the key space will increase more than 0.4 × 10^32^ ≈ 2^105^ approximately, which greatly extent the key space of original model.

Furthermore, by analyzing the sensitivity on small key differences, we focus on the newly introduced parameters *r* and *y*_0_, and change these parameters by 10^−16^. With different parameters, we can get different trajectories. Then we get the differences between these two trajectories, the results are shown in Fig. 2. Figure 2 indicates that the trajectories are completely different with a small difference of parameter after about 100 ns, which means that the newly brought in secret keys are rather sensitive.

**Complexity performances.** In this section, we will illustrate the complexity performance of our new model Eq. (2). The parameters are selected as *τ*_1,2_ = 25 ps, *θ*_1,2_ = 5 μs, Φ_1,2_ = −π/4, *u* = 1 ns, *T* = 30 ns, *U* = 0.5, *r* = 3.9999 and *y*_0_ = 0.1. In the experimentation, we use the Matlab R2014a to simulate a theoretical model. The five-order Dormand-Prince method is used here, where 1 ns is divided into 40 iterative steps.

*Chaotic trajectories.* Figure 3 shows the trajectories of model (2) for *β* = 4.5, indicating that our model is chaotic under these parameters and has good pseudorandom property. Furthermore, based on Fig. 3, the trajectory has a drift during the first 50 μs before *z*(*t*) enters the chaotic attractor. As time extends, the region of *z*(*t*) gradually approaches the chaotic attractor. This drift is not relevant to the delay time but induced by the model (2). After this drift, the trajectory will distribute in the whole chaotic attractor, showing that the model has a good ergodicity.

*Bifurcation and amplitude.* Figure 4 shows the bifurcation behavior of model (2) with bifurcation parameter *β*. When *β* = 0, the trajectory is a fixed point. As *β* increases, the trajectory will soon become chaotic. The simulation result indicates that our model can enter chaos region with a lower *β* than model (1), and other recently proposed systems in refs 14,21,23. This feature will enlarge the chaotic key space of parameter *β*. Figure 5 shows the amplitude in the asymptotic regime at each value of the bifurcation parameter. As *β* increases, the amplitude will gradually increase, resulting in a larger range of signal *z*(*t*).

*Approximate entropy.* ApEn was first proposed by Pincus^25^ to measure the complexity of time-series, which is closely related to the system distribution and correlation characteristics. The ApEn of the signals of this new model is shown in Fig. 6. As the gain coefficient *β* increases, the dynamical complexity will improve. As a result, ApEn will increase accordingly. From Fig. 6, we know that both ApEn of models (1) and (2) are positive correlated with *β*. Evidently, ApEn of this new model is larger than the ApEn of model (1) for the same coefficient *β*. For a relatively small *β*, the gap is larger. Furthermore, we compare the ApEn of our model with some proposed systems by setting *β* = 4.5. The ApEns of systems in refs 14,21,23 are 1.6373, 1.6510 and 1.7034, respectively, which are all smaller than the ApEn of our model. Therefore, our model is more complex than the original model (1) and other proposed systems in this sense.

*Permutation entropy.* PE is a complexity measure which was introduced in ref. 26 by Bandt *et al.* PE compares the size of some consecutive values in the sequence, and summed up different order types, then use Shannon’s entropy to measure the uncertainty of these ordering. PE is easily implemented and is computationally much faster than other comparable methods, such as Lyapunov exponents, while also being robust to noise^27^. As suggest in ref. 26, we select the ordinal pattern length *m* = 6, the embedding delay *D* = 2, and the length *N* of time series be 1.2 × 10^5^. Figure 7 compares the PE of model (1) and (2). From Fig. 7 we find out that the PE of our model (2) is larger than 0.99 when *β* > 4, indicating that the model (2) is relatively complex. By comparing the PE of model (2) and (1), the PE of model (2) is larger than the PE of model (1) when *β* ≦ 4. For *β* > 4, and the PE curves are very close with each other, because as *β* increases, the PE of original model (1) has already approached the ideal value 1. Although the gap is quite small, the PE has still been increased. Take *β* = 4.5 as an example, the PE of model (1) and (2) are 0.9884 and 0.9975 respectively. Comparing to the PE with other systems in refs 13,21,23. For *β* = 4.5, the PE of systems in these references are 0.9930, 0.9890 and 0.9910, respectively. Therefore, our model is evidently more complex.

**Time-delay concealment.** A secure optoelectronic delayed feedback system should conceal time-delay signature against other existing methods. Currently, ACF, DMI and PIA are the three most effective methods for identifying the time-delay. In this section, we will prove that our model (2) can effectively conceal the time-delay against these three methods.

*ACF method.* Figure 8 show the results of both models (1) and (2) by using ACF method for *β* = 4.5. For model (1), there is an obvious extrema at *t* = 30 ns, suggesting that the time-delay has been identified. While for model (2), there is no obvious extrema, showing that the time-delay has been concealed effectively.

The effect of parameter *β* on the time-delay concealment is further analyzed. First, we define the background *Q*_ACF_ as


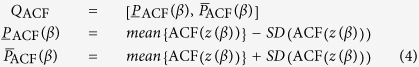


where *SD* is the standard deviation. For each *β*, *z*(*β*) is the output signal of model (2). The peak size at the relevant time-delay is defined as


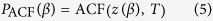


Figure 9 shows the size of the peak at the relevant time-delay *T* = 30 ns from the background (red lines) *Q*_ACF_ in ACF(*x*_1_). Figure 9 shows that the peaks are not apparent from the background *Q*_ACF_ once *β* increases to the approximate critical value 4.5. For *β* ≥ 4.5, the time-delay can be concealed using ACF method.

*DMI method.* Figure 10 shows the results of model (1) and (2) by using DMI method for *β* = 4.5. Figure 10 shows that there is an obvious peak at *t* = 30 ns, the exact time delay of model (1). While for model (2), there is no obvious extrema along the evolution, indicating that the time-delay cannot be identified.

We further analyze the influence of parameter *β* on the time-delay concealment. The background *Q*_DMI_ is defined as


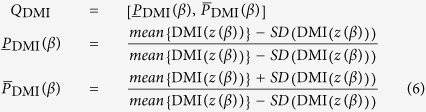


and the peak size at the relevant time-delay is defined as


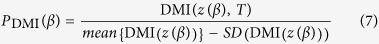


The peak size at the relevant time-delay *T* = 30 ns and the background (red lines) *Q*_DMI_ are shown in Fig. 11. From Fig. 11 we know that, when *β* ≥ 4.5, all the peak sizes are located in the background *Q*_DMI_, indicating that the time-delay will be concealed for *β* ≥ 4.5.

*PIA method.* Figure 12 shows the PE(*z*) of the embedding delay *T* is shown for *β* = 4.5 with ordinal pattern lengths *L* = 3, 4, 5, 6 and 7, respectively (The selection of *L* is suggest in ref. 26). Figure 12 suggests that there is no local extrema in PE(*z*), and that the time-delay can be concealed.

In order to analyze the influence of parameter *β* on the time-delay concealment, we define the background *Q*_PIA_ and the peak size at the relevant time-delay as


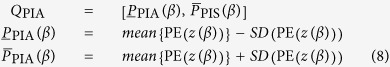



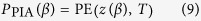


Figure 13 shows the peak size at relevant time-delay of 30 ns from the background (red lines) *Q*_PIA_ in PE(*x*_1_). As shown in Fig. 13, when *β* ≥ 4.5, all peak sizes are in the *Q*_PIA_ interval. This result shows that the time-delay is not available by PIA method.

In summary, the time-delay signal of model (2) can be effectively concealed using the ACF, DMI and PIA methods for *β* ≥ 4.5.

## Conclusion

In this paper, we propose a new switching parameter varying optoelectronic delayed feedback model with computer simulation. This model is switching between two parameter varying optoelectronic delayed feedback models based on chaotic pseudorandom number sequences. We use the trajectory diagram, bifurcation diagram, approximate entropy and permutation entropy to illustrate its complexity. The results show that this model is more complex than the original model (1). We prove that our new model can conceal the time delay effectively against the ACF, DMI and PIA methods. Furthermore, some tunable parameters are brought in, which can extent the key space to resist brute-force attack.

As we know, chaotic systems have a widely use in many different kinds of fields, especially in cryptography. This paper discusses and proposes a new chaotic model, which has a high dynamical complexity, large key space, and proper conceal of time-delay compared to other common methods. All these characteristics show that our chaotic model is relatively secure to be applied in chaotic cryptography, including chaotic secure communication and chaotic pseudorandom sequence generator. For future work, we will study the applications based on this new chaotic model.

## Additional Information

**How to cite this article**: Liu, L. *et al.* A new switching parameter varying optoelectronic delayed feedback model with computer simulation. *Sci. Rep.* **6**, 22295; doi: 10.1038/srep22295 (2016).

## Figures and Tables

**Figure 1:**
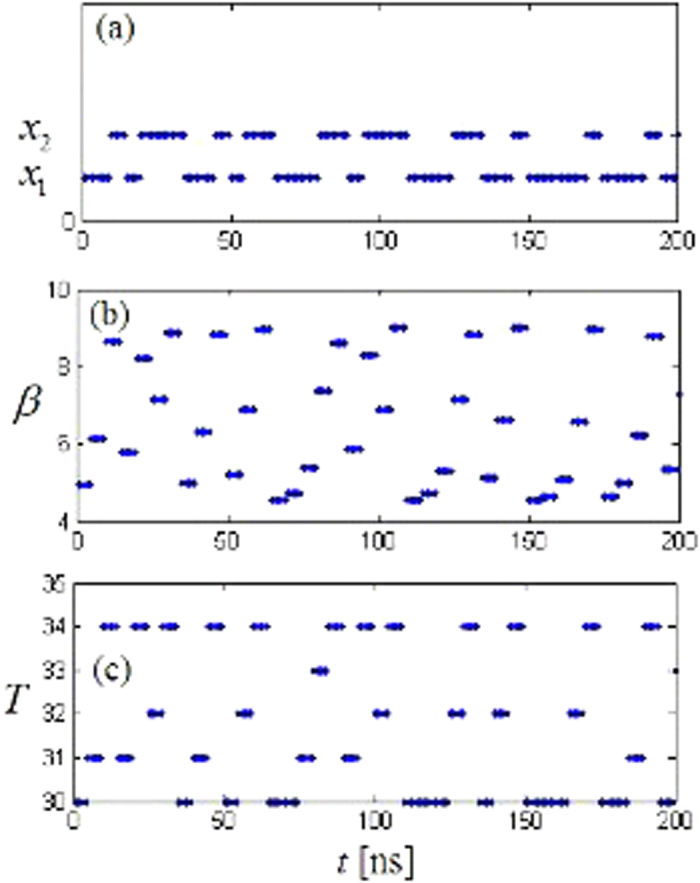
The transfer signal **(a**) *z*(*t*); (**b**) *β*(*t*); and (**c**) *T*(*t*).

**Figure 2:**
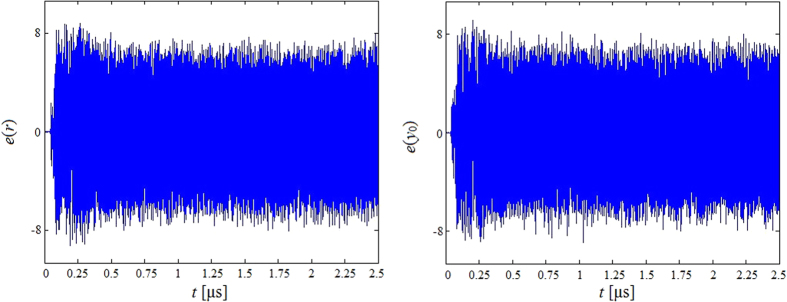
The differences between the trajectories with small difference of parameters (**a**) *r*; (**b**) *y*_0_.

**Figure 3:**
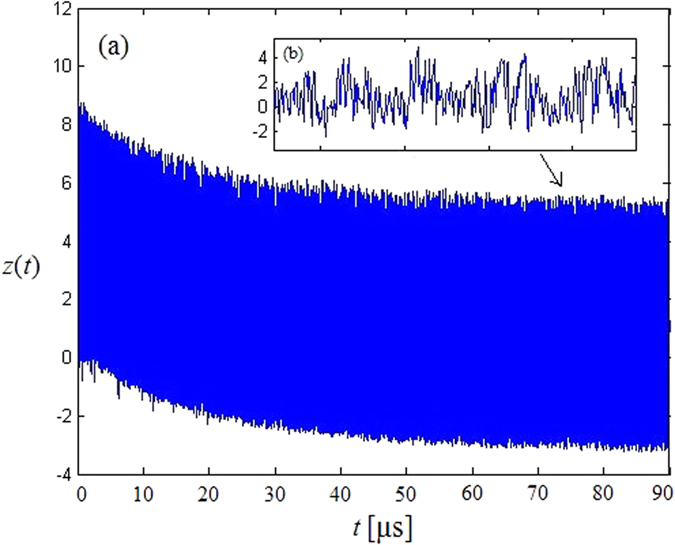
(**a**) The trajectories of our new model; (**b**) Enlargement of (**a**).

**Figure 4:**
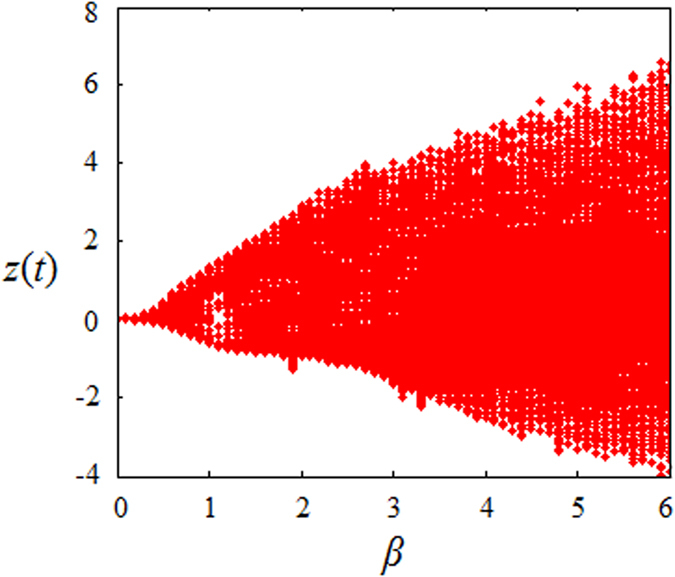
Bifurcation diagram of model (2).

**Figure 5:**
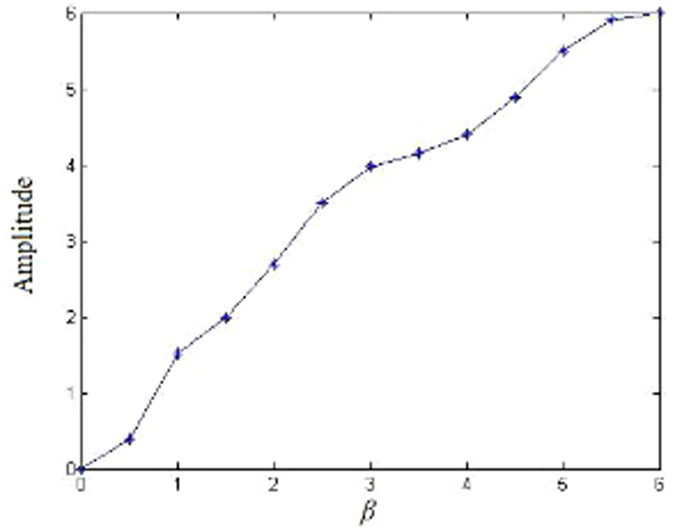
Amplitude at each value of the bifurcation parameter of model (2).

**Figure 6:**
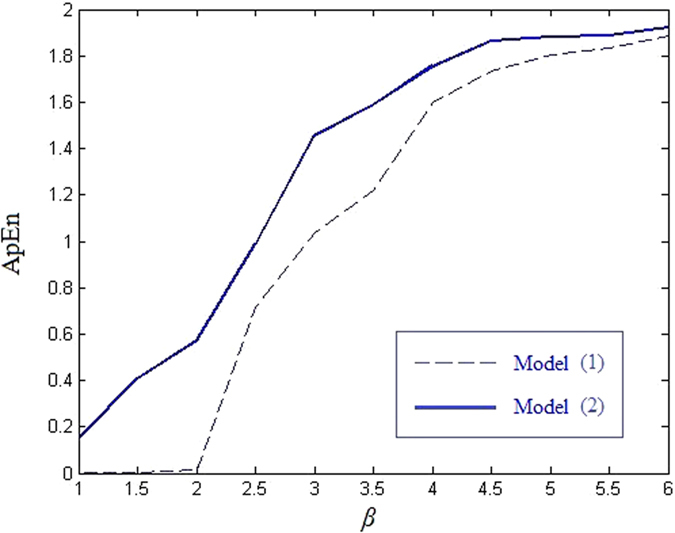
Approximate entropy comparison.

**Figure 7:**
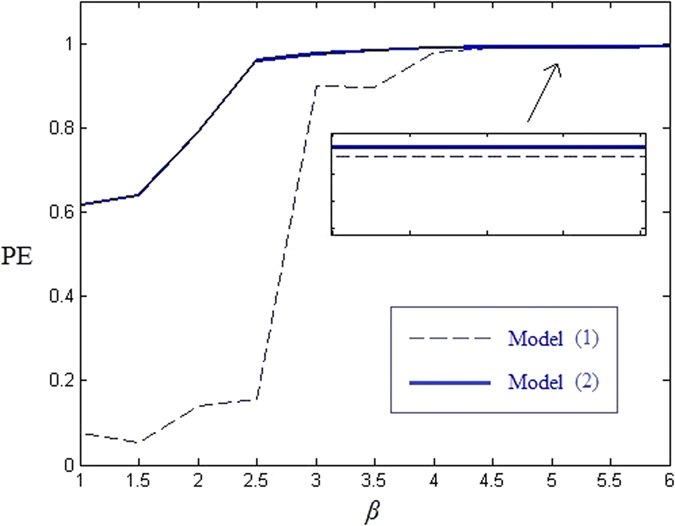
Permutation entropy comparison.

**Figure 8:**
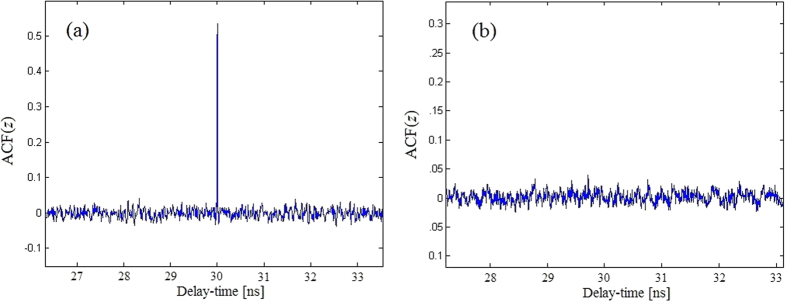
ACF(*z*). (**a**) Model (1); (**b**) Model (2).

**Figure 9:**
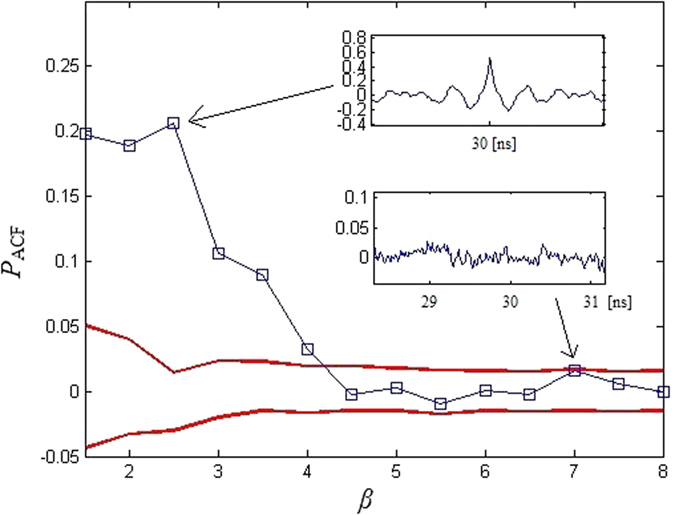
Value of the peaks in ACF(*z*) at *T* = 30 ns. The red lines correspond to the background *Q*_ACF._

**Figure 10:**
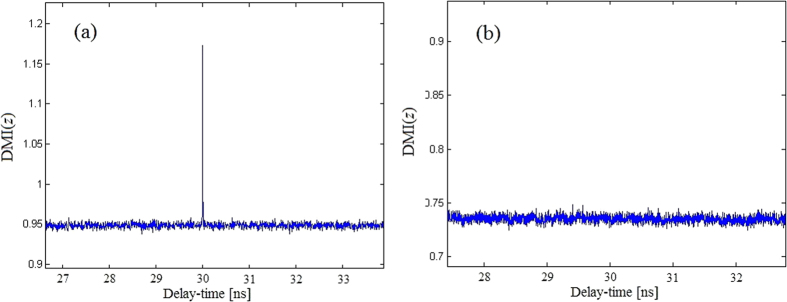
DMI(*z*). (**a**) Model (1); (**b**) Model (2).

**Figure 11:**
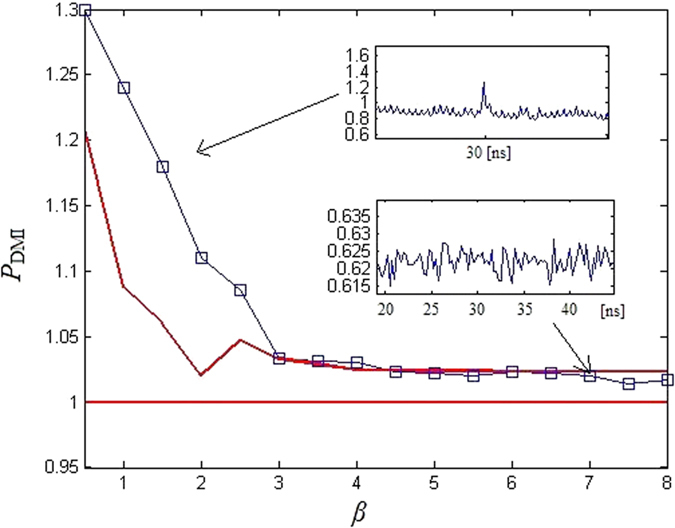
Value of the peaks in DMI(*z*) at *T* = 30 ns. The red lines correspond to the background *Q*_DMI_.

**Figure 12:**
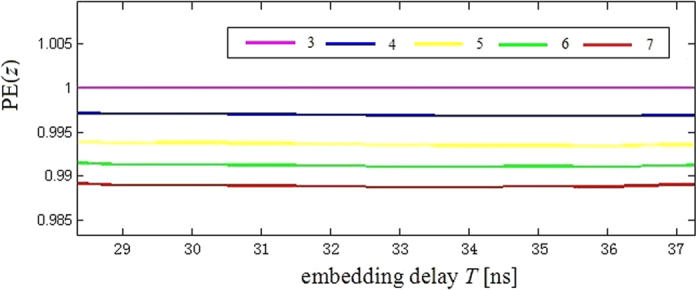
PE(*z*) with ordinal pattern lengths *L* = 3, 4, 5, 6 and 7, respectively.

**Figure 13:**
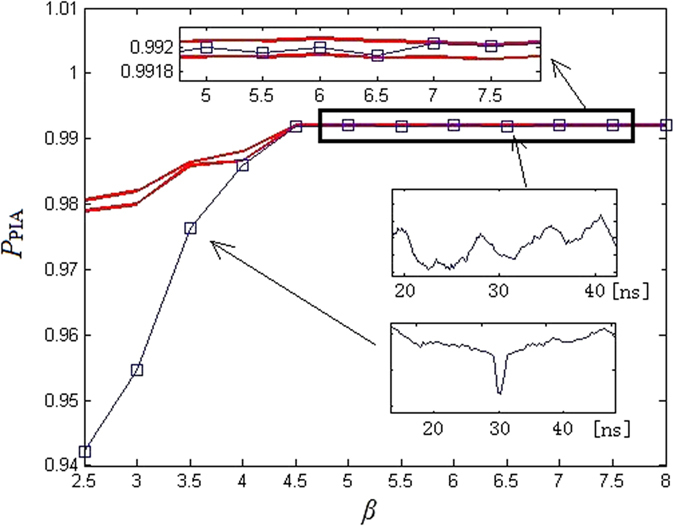
Value of the peaks in PE(*z*). The red lines correspond to the background *Q*_PIA_.
